# Role of Symbiotic Bacteria in the Growth and Development of the Sunn Pest, *Eurygaster integriceps*

**DOI:** 10.1673/031.013.9901

**Published:** 2013-09-30

**Authors:** Maryam Kafil, Ali Reza Bandani, Martin Kaltenpoth, Seyed Hossein Goldansaz, Seyed Mehdi Alavi

**Affiliations:** 1 Department of Plant Protection, University of Tehran, Karaj, Iran; 2 Max Planck Institute for Chemical Ecology, Insect Symbiosis Research Group, Hans-Knoell-Str. 8, 07745 Jena, Germany; 3 Department of Plant Biotechnology, National Institute for Genetic Engineering and Biotechnology (NIGEB), P.O.Box: 14155-6343, Tehran, Iran

**Keywords:** antibiotic effect, egg sterilization, Hemiptera, norfloxacin, symbiont

## Abstract

The Sunn pest, *Eurygaster integriceps* Puton (Hemiptera: Scutelleridae), is the most important pest of wheat and barley in wide areas of the world. Different aspects of the insect's life history have been studied, but to date nothing is known about their microbial symbionts. Here, the contribution of symbiotic bacteria to the fitness of the bug was investigated by combining two different approaches to manipulate the host's microbial community: the supplementation of antibiotics into the insects' diet and egg surface sterilization. First, bacteria cultured from gut homogenates were subjected to antibiotic screening tests using 20 different antibiotics. Norfloxacin was the most effective antibiotic, with the greatest inhibition zone among all antibiotics tested. Feeding norfloxacin to adult *E. integriceps* individuals significantly impaired growth and development of the offspring in a dose-dependent manner, i.e., higher antibiotic doses increased the negative effects on nymphal growth and development. Total developmental time from first nymphal instars to adult emergence in control animals was 30.1 days, but when adults had been offered diets with 10, 20, and 30 µg antibiotic per mg diet, the offspring's developmental time was prolonged to 32.8, 34.0, and 34.8 days, respectively. In the highest two doses of norfloxacin, all of the nymphs died before reaching the fifth nymphal instar. Similar results as for the antibiotic treatment were obtained when egg surface sterilization was used to manipulate the microbial community of *E. integriceps*. These results indicate that bacterial symbionts play a crucial role in the successful development of the host.

## Introduction

Insects are able to occupy a vast array of habitats, including marine and terrestrial environments (Grimaldi and Engel 2005). A major factor that contributed to the insects' success in occupying a variety of habitats is their adaptation to feed on different diets (Dow 1986). This flexibility of feeding habits is often provided by endosymbionts, and some estimates suggest that up to 20% of all insect species harbor obligate symbiotic microorganisms (Ishikawa 2003; Moran 2006).

Herbivorous insects are facing an imbalanced diet because of excess carbohydrates in comparison with those insects feeding on a carnivorous diet containing sufficient amounts of nitrogenous compounds. One strategy to overcome such limitation is to change the feeding habit towards a more predatory life-style (Denno and Fagan 2003). However, feeding on such diverse food sources needs physiological and morphological specialization. Thus, a different adaptation is to exploit the metabolic potential of endosymbionts, microorganisms that live as symbionts in the insect body. These symbionts enable insects to feed on food resources that are rich in some nutrients and lack or are scarce in some others. Such adaptation is seen in aphids feeding on plant saps (xylem or phloem), which are rich in carbohydrates but contain only trace amounts of some nitrogenous compounds (notably the essential amino acids) that are essential for the insect's growth and development (Baumann 2005).

Earlier studies have shown that some insects, such as plant sap or blood sucking insects and some ant species, contain specialized cells filled with bacteria called bacteriocytes. These insect cells are intercalated between midgut cells (as in carpenter ants) or they form organ-like structures consisting of cell clusters located adjacent to the gut or fat body (as in aphids or cockroaches) (Baumann 2005). Within the bacteriocytes, the bacteria either are located in the cytosol (*Blochmannia*, *Wigglesworthia*, *Baumannia*) or enclosed in the cell vacuoles (*Buchnera*) (Baumann 2005).

Primary endosymbionts are usually required for host survival by contributing to the hosts' diet by providing nutrients (Feldhaar 2011). Experimental studies and genomic analyses have shown that bacterial symbionts can provide essential amino acids to phloem-feeding insects as well as to generalists (e.g., *Buchnera aphidicola* in *Acyrtosiphon pisum* and *Blochmannia floridanus* in *Camponotus floridanus*), or B vitamins to blood-sucking insects (e.g., *Wolbachia* in *Cimex lectularius* and *Wigglesworthia* in *Glossina*) (Zientz et al. 2006; Brownlie et al. 2009; Gunduz and Douglas 2009; Hosokawa et al. 2010). Furthermore, fungal symbionts may be involved in sterol synthesis of some insects, and many wood-feeding insects rely on symbiotic microorganisms that aid in the degradation of cellulose (see Douglas 2009 for review).

Mutualistic associations between bacteria and insects are categorized into primary (obligate) and secondary (facultative) symbionts (Baumann 2005). The symbiosis between aphids and *Buchnera aphidicola* (Munson) represents the best known relationship between insects and bacteria, which is an obligatory association in which neither partner can live without the other. *Buchnera* provides essential amino acids to the host aphid and its transmission is vertically (transovarially) from one generation to the next, thus, leading to an intimate relationship. The elimination of the mutualistic bacteria by antibiotic treatment severely affects aphid growth, development, and fecundity, with aposymbiotic aphids growing poorly and producing few or no offspring (Baumann et al. 1995; Douglas 1998; Sauer et al. 2000). Secondary symbionts, which are not required for host survival and their existence is not confined to the bacteriocytes, can affect the fitness of the insect host in different ways, e.g., by providing heat stress tolerance, compensation for loss of *B. aphidicola* in aphids, resistance to parasitoid wasps, or resistant to pathogens (Montllor et al. 2002; Koga et al. 2003; Oliver et al. 2003; Dillon and Dillon 2004; Scarbourough et al. 2005; Russell and Moran 2006; Prado et al. 2009).

Several studies have shown that a large diversity of symbiotic relationships exist between Hemipteran insects and bacteria. In most cases, the symbionts reside in the gut lumen (Reduviidae) or in the gastric caeca (e.g., Pentatomidae, Plataspidae, and Alydidae) (Abe et al. 1995; Fukatsu and Hosokawa 2002; Prado and Almeida 2009). These bacteria are usually vertically transmitted through infection of materials associated with the egg masses or fecal pellets (Buchner 1965; Fukatsu and Hosokawa 2002; Prado et al. 2006; Prado and Almeida 2009).

The functional role of bacterial symbionts in Hemiptera has been investigated for a long time, and several studies showed that many symbionts play a nutritional role (Buchner 1965; Abe et al. 1995; Douglas 1998; Fukatsu and Hosokawa 2002; Hosokawa et al. 2005; Hirose et al. 2006; Prado et al. 2006; Kaltenpoth et al. 2009; Prado and Almeida 2009; Kaiwa et al. 2011). The presence of crypts in the midgut of Scutellerid bugs has been known since the early 20^th^ century (Glassgow 1914), and the presence of bacterial symbionts in these crypts has been reported recently (Kaiwa et al. 2011). Similar crypts were also shown to exist in the gut of the Sunn pest, *Eurygaster integriceps* Puton (Hemiptera: Scutelleridae) (Mehrabadi et al. 2012).

Despite the agricultural importance *E. integriceps*, which is the most serious insect pest in cereal crops in a wide area of the world, especially in the countries from the Middle East to southern Europe, including Afghanistan, Iran, Iraq, Jordan, Pakistan, Syria, Lebanon, Turkey, and Spain (Moore 1998), there is no information about its associated microorganisms. Knowledge about the bacterial symbionts associated with *E. integriceps* is a key step in developing novel control strategies for this species. Microscopic studies have already revealed the presence of bacterial symbionts in the fourth ventriculus (fourth stomach) (Mehrabadi et al. 2012). Thus, as an important step is to investigate the function of these symbionts in the insect's biology, two methods of symbiont manipulation, antibiotic treatment and egg surface sterilization, were used to elucidate the effects of the *E. integriceps*-associated microorganisms on the insect's growth and development.

## Materials and Methods

### Insect Rearing

The adult *E. integriceps* were collected from wheat and barley farms (Karaj, Iran, 35° 43′ 59.95″ N, 50° 17′ 08.19″ E) and transferred to the laboratory. The insects were reared at 25 ± 5° C and 60 ± 10% RH, with a 16:8 L:D photoperiod, as described by Allahyari et al. (2010).

### Chemicals

All chemicals were purchased from Merck (www.merck.com) except for norfloxacin, which was obtained from Sigma-Aldrich (www.sigmaaldrich.com).

### Extraction of the insects' guts and bacterial cultivation

Adult female *E. integriceps* were placed at 20° C for 5 min. Insects were surface-sterilized using ethanol (75%) and dissected using sterilized forceps, and their whole guts were removed. The guts were homogenized in 500 µl sterile saline solution (0.85% NaCl) by glass homogenizer. This homogenate was used for cultivation of gut bacteria in super optimal broth medium based on Visotto et al.(2009). This medium consisted of 20 g bactotryptone, 5 g bacto yeast extract, 0.5 g NaCl, 2.5 mL 1M KCl, and 900 mL sterile distilled water (Hanahan 1983). The medium was inoculated with gut bacteria, which were then allowed to grow for 4 hr at 28° C (the optimum temperature for the insect growth) in a shaker incubator at 170 rpm. These grown bacteria were used for subsequent antibiotic screening tests.

### Antibiotic screening tests

Antibiotic screening tests were carried out *in vitro* based on Visotto et al. (2009). Gut bacteria grown in the super optimal broth medium (100 µl) were inoculated in Mueller Hinton agar medium (Atlas 2004). This medium consisted of 30.0% beef infusion, 1.75% casein hydrolysate, 0.15% starch, 1.7% agar, and a pH adjusted to 7.0. The antibiotic discs were placed with sterilized forceps on the surface of the medium containing the gut bacterial suspension. The discs contained known concentrations of 20 antibiotics (Table 1), and their application followed the manufacturer's recommendations (Tadbir Fan Azma, http://www.tadbirkit.ir/). The gut culture plates were incubated overnight at 28° C. Then, inhibition zones were measured according to Madigan et al. (2000).

**Table 1.**
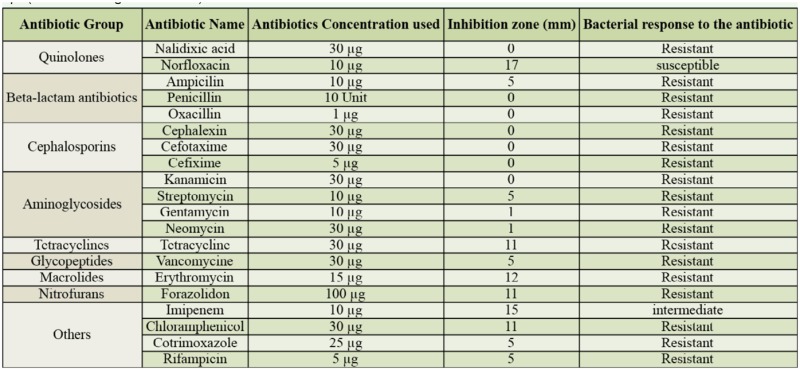
Antibiotics and their concentrations used in the antibiotics screening test, and their effect on gut bacteria of *Eurygaster integriceps* (based on Madigan et al. 2000).

### Effect of gut bacteria on insect development

Norfloxacin, the most effective antibiotic against the gut bacteria under *in vitro* conditions, was subjected to another set of tests to assess its effectiveness in insect growth and development based on Visotto et al. (2009). This antibiotic was incorporated into the insect diet at concentrations of 0 (as control), 10, 20, 30, 40, and 50 µg/mg diet. Artificial diet containing different concentrations of antibiotics were prepared as described by Saadati and Bandani (2010). To reduce the side-effects of antibiotics on the insect itself, the adult *E. integriceps* were treated with antibiotics, and the effects on the offspring were recorded (egg hatching success and nymphal growth and development). These F1 individuals were still aposymbiotic, but had never been in direct contact with the antibiotic (De Vries et al. 2004).

Adult insects (30 females per antibiotic concentration) were allowed to feed on artificial diets containing different concentrations of norfloxacin for 2 days as described by De Vries et al. (2004). Control insects were fed on artificial diet without antibiotic. Then, the insects were allowed to lay eggs, and thirdday-eggs were collected. Egg hatching success, developmental time of each nymphal stage, and their mortality rate were recorded by daily observations. Also, adult weight (0–24 hr old) was recorded. Diets were replaced with fresh diet every 2–3 days. The insect growth condition was as described before.

### Effect of egg surface sterilization on *E. integriceps* growth and development

The experiment on the effect of egg surface sterilization on *E. integriceps* growth and development was done based on Kaltenpoth et al. (2009). Briefly, 500 eggs laid on the same day were collected and assigned into 2 equal groups, with 1 group treated (egg surface sterilized) and the other group left as an untreated control. Two days before hatching, the egg mass of the treatment group was treated with 95% ethanol for 5 min and then submerged in bleach (12% NaOCl) for 15 sec. The residual bleach was washed off carefully with sterile water, and the eggs were dried on filter paper. The control eggs were only rinsed in distilled water. The measured parameters were percent of egg hatching success, nymphal developmental time, nymphal mortality, percentage of adult emergence, and adult weight (0–24 hr).

### Statistical analysis

Data on developmental time, mortality, and weight were analyzed by one-way ANOVA followed by Duncan's multiple range tests using SAS program (SAS Institute 2003). Data obtained from sterilization of eggs were analyzed using SPSS (SPSS 2007).

## Results

### Antibiotic screening assays

Based on the antibiotic screening tests, all tested 20 antibiotics were separated into 4 groups according to the inhibition zone they produced: 0.0–0.5 cm (first group), 0.5–1.0 cm (second group), 1.0–1.5 cm (third group), and > 1.5 cm (fourth group) (Table 1). Seven antibiotics were placed in the first group that had the least activity against gut bacteria: Cefixime, Kanamycin, Cephalexin, Oxacillin, Penicillin, Nalidixic acid, and Cefotaxime. Five antibiotics were in the second group that had moderate activity against gut bacteria: Ampicilin, Streptomycin, Co trimoxazole, Rifampin, and Vancomycin. The third group contained 6 antibiotics that produced a good inhibition zone against gut bacteria: Gentamycin, Furazolidon, Erythromycin, Chloramphenicol, Tetracycline, and Neomycin. Finally, only 2 antibiotics (Imipenem and Norfloxacim) were placed into fourth group. Since only 1 antibiotic (Norfloxacim) produced a very good inhibition zone against gut bacteria in the *in vitro* assay, this antibiotic was chosen to be incorporated into the diet to assess its activity on the insect growth and development.

**Table 2.**
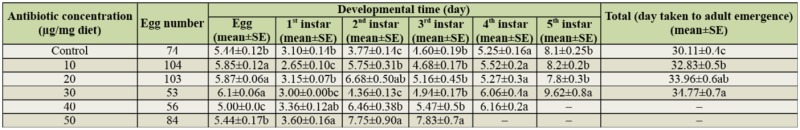
The effect of different concentrations of norfloxacin on *Eurygaster integriceps* growth, development, and adult weight. The adult *E. integriceps* were given antibiotics in their diet, and then they were allowed to lay eggs. The growth and development of the nymphs emerged from these eggs were monitored, and their mortality at each stage and adult weight (0–24 hr) were recorded. In each column, means ± SE followed by the same letter indicate no significant difference (*p* < 0.05) between data based on Duncan's test.

### The effect of norfloxacin on insect growth and development

The duration of different growth stages of *E. integriceps* offspring from females that had been kept on 5 different concentrations of antibiotic is shown in Table 2. Embryonic development (time taken from egg laying until hatching) in the control was 5.44 days, but in treatments it was prolonged (5.85, 5.87, 6.1, 5.0, and 5.44 days at concentrations of 10, 20, 30, 40, and 50 µg antibiotic per mg diet, respectively). ANOVA showed that there were significant differences among treatments (*F*= 20.8, df = 5, *p* < 0.0001), with the greatest delay in embryonic development at 30 µg antibiotic per mg diet (Table 2). Surprisingly, embryonic development at 50 µg antibiotic per mg diet was the same as in untreated control individuals.

**Figure 1.**
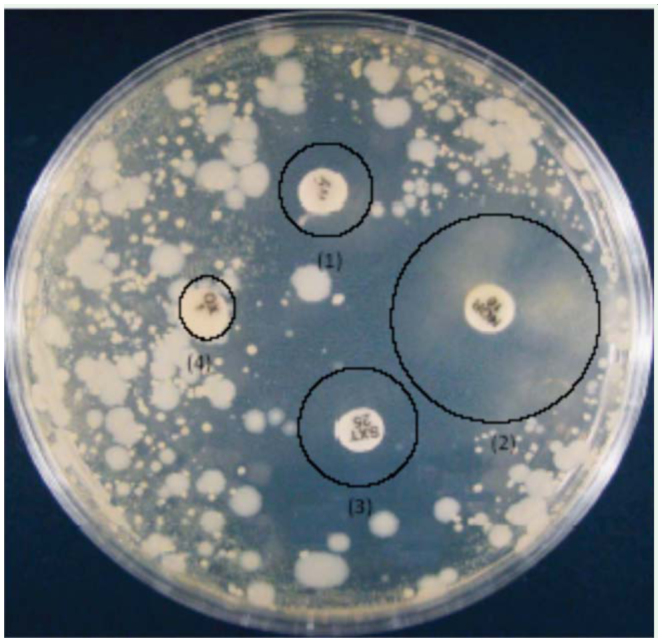
Comparison of inhibition zones produced by different antibiotics. The antibiotics shown are as follows: (1) Ampicillin, (2) Norfloxacin, (3) Cotrimoxazol, (4) Oxacillin. High quality figures are available online.

**Figure 2.**
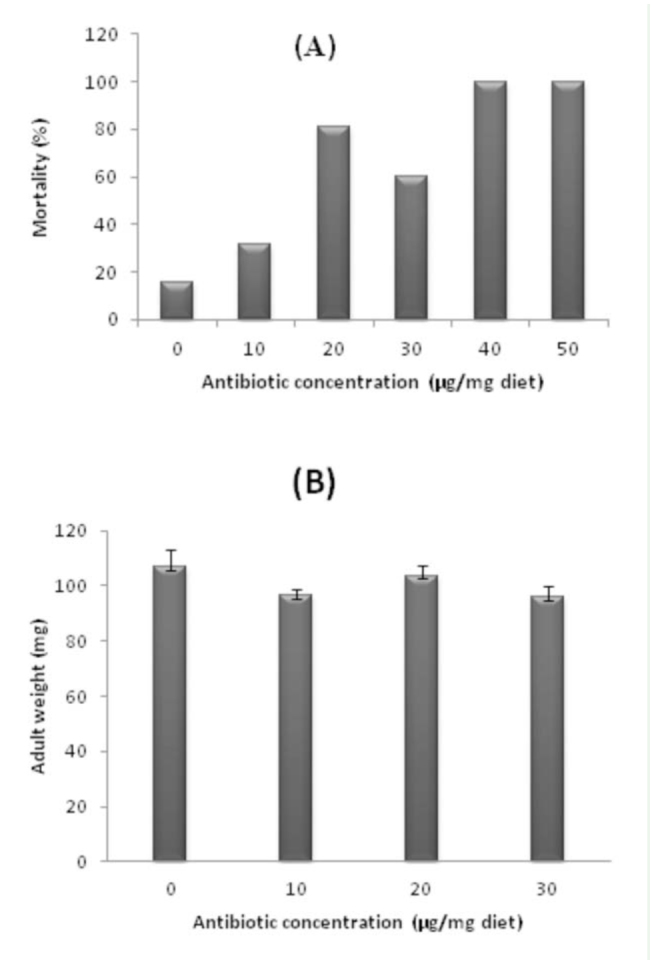
Effects of different antibiotic concentrations on *Eurygaster integriceps* mortality percentage (A) and adult weight (B). High quality figures are available online.

Nymphal development was also affected by prior incorporation of the antibiotic into the mother's diet. Significant differences among treatment groups were found in the duration of first (*F* = 5.06, df = 5, *p* = 0.0002), second (*F* = 15.3, df = 5, *p* < 0.0001), and third nymphal instars (*F* = 5.94, df =5, p < 0.0001). For example, the developmental time in the third nymphal instar in control individuals was 4.6 days, while it was 5.47 and 7.83 days in 40 and 50 µg antibiotic per mg diet, respectively.

In the highest two doses (40 and 50 µg antibiotic per mg diet) all died before reaching the fourth or fifth nymphal instar (Figure 2A). The effect of antibiotics on insect mortality was dose dependent. With increasing antibiotic dose, mortality also increased. Total developmental time from first nymphal instars to adult emergence in the control was 30.1 days, but when adults had been offered diets with 10, 20, and 30 µg antibiotic per mg diet, the offspring's developmental time was prolonged to 32.8, 34.0, and 34.8 days, respectively (*F* =10.5, df = 4, *p* < 0.0001). Adult weight did not show significant differences between control and treatment individuals, although there was a tendency towards lower adult weight in the antibiotic-treated groups (Figure 2B).

### The effect of egg surface sterilization on *E. integriceps* growth and development

**Figure 3.**
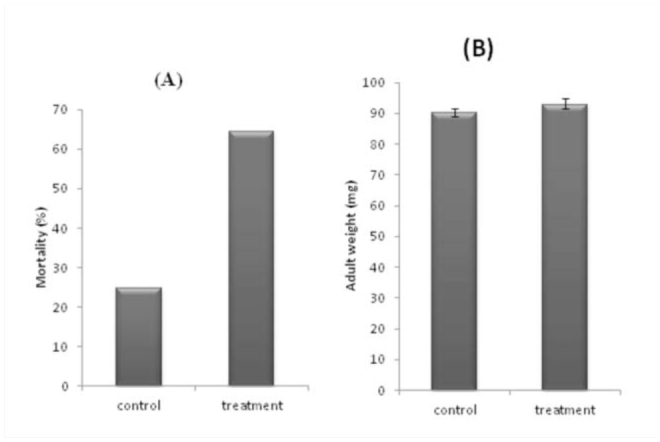
Effects of egg surface sterilization on mortality (A)and adult weight (B) of *Eurygaster integriceps*. High quality figures are available online.

Egg mass sterilization affected developmental time and mortality (Table 3). The effect of egg surface sterilization was more pronounced in embryonic development and early stages of post-embryonic development, i.e., developmental time of the first and the second nymphal instars were significantly different from that of control insects. However, there were no significant differences between control and treatments in the last developmental stages (third, fourth, and fifth instar nymphs, *p* > 0.05). Also, there were no significant differences in weight of controls and treated insects (Figure 3B). However, mortality during nymphal development until adult emergence was strongly elevated after egg surface sterilization as compared to the control group (Figure 3A).

**Table 3.**
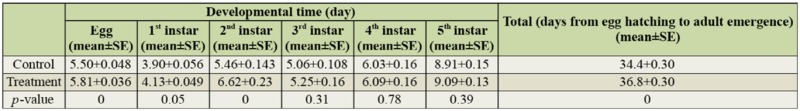
The effect of egg surface sterilization on *Eurygaster integriceps* nymphal growth and development and the adult weight. The egg surface was sterilized, and the insect growth and development was monitored until adult emergence. Nymphal duration, total days taken to adult emergence, and adult weight (mg) were determined.

## Discussion

In the current study, the role of bacterial symbionts in growth and development of *E. integriceps* was investigated. It has long been established that bacteria associated with insects can be involved in food digestion, providing nutrition, detoxification, pheromone production, regulation of pH, synthesis of vitaminsand sterols, temperature tolerance, resistance against pathogens and parasitoids, and even modifying the use of host plants by phytophagous insects (Dillon and Dillon 2004; Genta et al. 2006; Feldhaar 2011; Ferrari and Vavre 2011), thereby affecting insect development, defense against natural enemies, immunity, reproduction, and speciation (Hurst and Werren 2001; Braendle et al. 2003; Dillon and Dillon 2004; Koropatnick et al. 2004; Backhed et al. 2005; Baumann 2005; Macdonald and Monteleone 2005).

In order to study the role of symbionts on the insect's fitness, the microbial community of *E. integriceps* individuals were manipulated, and the effects on development, mortality, and adult weight were assessed. Thus, the effects of a diverse number of antibiotics were investigated *in vitro*. This antibiotic screening test showed that 20 tested antibiotics fell into 4 groups based on the inhibition zone they produced. Among the tested antibiotics, norfloxacin yielded the largest inhibition zone in antibiotic screening tests. Norfloxacin has been reported to be a broad-spectrum antibiotic that is active against both Gram-positive and Gram-negative bacteria. It functions by inhibiting DNA gyrase, a type II topoisomerase, and topoisomerase IV. These enzymes are necessary for separation of bacterial DNA, thereby inhibiting cell division (Drlica and Zhao 1997). This antibiotic is occasionally used to treat urinary tract infections (Rafalskky et al. 2006).

Norfloxacin was orally administrated to the adult insects to reduce the abundance of symbiotic bacteria in the gut in order to study the role of the symbionts on the fitness of *E. integriceps*. The results showed a dose-dependent effect of the antibiotic treatment on the offspring of the treated individuals, with development and survival probabiliy of *E. integriceps* individuals being significantly impaired at high doses of antibiotic. These results suggest an important functional role of these symbionts for the insect's growth and development, as well as indicate the obligate nature of the host-symbiont association. However, antibiotic treatments did not affect adult weight, as there were no significant differences in adult weight between aposymbiotic and control individuals. Similar to our finding, De Vries et al. (2004) observed that antibiotical elimination of *Erwinia* from *Frankliniella occidentalis* retarded the host's developmental time. Visotto et al. (2009) showed that increased concentrations of tetracycline caused mild increases in *Anticarsia gemmatalis* mortality. Another important result was that early nymphal growth (first, second, and third instars) of the *E. integriceps* was more affected by antibiotic treatment than the later stages ( the fourth and fifth nymphal stages). Similar results were obtained by Tada et al. (2011), who found that experimental sterilization of *Nezara viridula* eggs resulted in severe nymphal mortality. They also showed that early nymphal instars of the insect were more affected by egg sterilization than later stages, which agrees with our findings on *E. integriceps*.

In the present study, egg surface sterilization was done as an additional method to reduce symbionts load in order to exclude the possibility that the antibiotic treatment directly affected the insects themselves. The results confirmed those from the antibiotic treatment, showing prolonged developmental times and higher mortality in the aposymbiotic group. As in the antibiotic treatment, adult weight was not affected when egg surface sterilization was performed. The same result was found when *Pyrrhocoris apterus* eggs were sterilized by Kaltenpoth et al. (2009). They found that elimination of symbionts by eggsurface sterilization lead to retarded growth and a significant increase in mortality of the treated insects. Similarly, Abe et al. (1995) showed that individuals from surface sterilized egg masses of *Plautia stali* did not reach the adult stage. Also, it has been shown that *Acrosternum hilare* development time, survivorship, and reproductive success were negatively affected by egg surface sterilization (Prado and Almeida 2009).

In true bugs (suborder Heteroptera), three mechanisms have been reported for symbiont transmission, including coprophagy, bacteria-containing capsules, and egg smearing (Buchner 1965; Abe et al. 1995; Fukutsa and Hosokawa 2002). For scutellerid bugs, Kaiwa et al. (2010) studied *Cantao ocellatus*, and by egg surface sterilization experiments confirmed that the bacterium is transmitted to stink bug nymphs via the egg surface. Similarly, Pentatomid bugs transmit symbiotic bacteria vertically to their offspring by smearing cells on the surface of the egg masses during oviposition. Aposymbiotic (symbiontfree) first instar bugs acquire the symbionts by probing the egg surface, which is the main reason why these nymphs remain aggregated around the egg clutch and do not feed much during the first nymph instar (Buchner 1965; Abe et al. 1995; Prado et al. 2006).

In conclusion, the results of our study on *E. integriceps* indicated that, as in several other hemipteran taxa, aposymbiotic nymphs produced either by antibiotic administration or egg surface sterilization exhibited retarded growth and/or increased mortality, suggesting that the symbionts play a crucial role for the host insects. Thus, the gut symbiotic bacteria could possibly be used as a target for controlling this devastating crop pest.
